# Early-d-metal Janus MXenes as near-thermoneutral hydrogen evolution electrocatalysts: role of anion identity and d-band asymmetry

**DOI:** 10.1039/d6ra02989b

**Published:** 2026-05-26

**Authors:** Shrestha Dutta, Rudra Banerjee

**Affiliations:** a Department of Physics and Nanotechnology, SRM Institute of Science and Technology Kattankulathur Tamil Nadu 603203 India rudrab@srmist.edu.in

## Abstract

Identifying earth-abundant alternatives to platinum for the hydrogen evolution reaction (HER) remains a central challenge in electrocatalysis. Janus MXenes—two-dimensional carbides or nitrides with chemically distinct metal sublattices—break the mirror symmetry of conventional MXenes and create an electronic non-equivalence between the two oxygen-terminated surfaces that is inaccessible in symmetric structures. Here we exploit this asymmetry through a spin-polarised GGA + *U* density functional theory screening of six O-terminated Janus MXenes M_1_M_2_XO_2_ (M_1_/M_2_ = Ti/Zr, V/Nb, Cr/Mo; X = C, N), constructed as a 3 × 2 factorial series that disentangles d-electron count, 3d/4d orbital extent, and anion electronegativity. Among the thermodynamically stable candidates, only the group-5 VNb pair achieves near-thermoneutral hydrogen binding: VNbCO_2_ (Δ*G*_H*_ = −0.03 eV) and VNbNO_2_ (−0.11 eV) both satisfy |Δ*G*_H*_| ≤ 0.3 eV, whereas the group-4 TiZr systems under-bind and the group-6 CrMo nitride exhibits anomalous under-binding driven by exchange splitting of the Cr d-states. A site-resolved d-band centre analysis reveals that the 3d-metal *ε*_d_ correlates strongly with Δ*G*_H*_ across the non-magnetic systems (*R* = −0.91) but loses predictive power when spin polarisation is significant. The anion sublattice acts as a secondary compositional lever—shifting Δ*G*_H*_ by 0.08 eV within the VNb pair while triggering a 0.67 eV destabilising swing in the magnetic CrMo pair—demonstrating that its tuning capacity is contingent on the metal pair having first placed the system near the volcano apex. These results establish that optimal HER performance in early-d-metal Janus MXenes requires a non-magnetic, spatially asymmetric 3d/4d pair at intermediate d-filling, and identify the VNb combination as a computationally promising candidate for noble-metal-free electrocatalysis.

## Abstract 2

Identifying earth-abundant alternatives to platinum for the hydrogen evolution reaction (HER) remains a central challenge in electrocatalysis.

## Graphical abstract

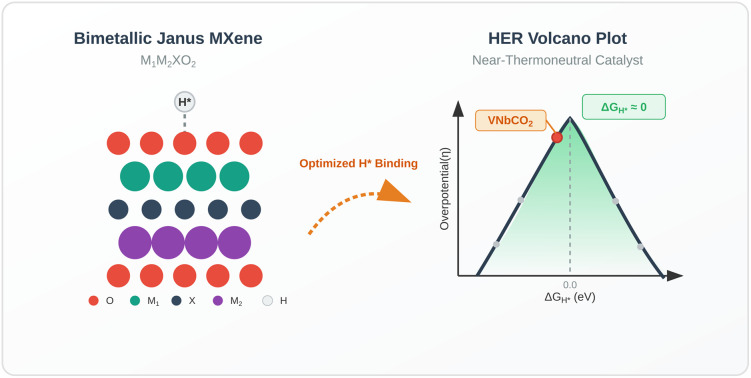

## 1 Introduction

The efficiency of electrochemical hydrogen production is governed by the strength of hydrogen binding at the catalyst surface. According to the Sabatier principle, an optimal hydrogen evolution reaction (HER) catalyst must bind hydrogen strongly enough to facilitate the Volmer step—proton–electron transfer to the surface—yet weakly enough to permit facile H_2_ desorption *via* the Heyrovsky or Tafel pathway.^1^ This trade-off is captured by a single thermodynamic descriptor, the Gibbs free energy of hydrogen adsorption (Δ*G*_H*_), which defines a volcano-shaped relationship with the exchange current density; the apex at Δ*G*_H*_ ≈ 0 marks the thermoneutral optimum.^1,2^ Platinum sits near this apex (Δ*G*_H*_ ≈ −0.09 eV), accounting for its benchmark status,^1^ but the scarcity and cost of platinum-group metals preclude their deployment at the terawatt scale demanded by industrial water electrolysis.^3,4^ A sustained search for earth-abundant alternatives that satisfy the Nørskov criterion |Δ*G*_H*_| ≤ 0.3 eV therefore remains a central challenge in electrocatalysis.^2,4^

Two-dimensional transition-metal carbides and nitrides (MXenes), first synthesised by selective etching of Ti_3_AlC_2_ in 2011,^5^ have attracted considerable attention as candidate HER electrocatalysts.^6,7^ With the general formula M_*n*+1_X_*n*_T_*x*_ (M = early transition metal; X = C or N; *T*_*x*_ = surface termination), MXenes combine metallic conductivity with a hydrophilic, chemically tunable surface.^6,8^ Among the accessible terminations, the O-terminated surface is of particular significance for HER because the surface oxygen atoms serve as the primary adsorption centres for hydrogen.^2^ Yet this very feature poses a persistent challenge: most O-terminated MXenes bind hydrogen too strongly, yielding Δ*G*_H*_ ≪ 0 and consequent site poisoning that limits catalytic turnover.^9^

Janus MXenes—structures in which the two outer metal sublattices are occupied by chemically distinct transition metals—offer a route out of this over-binding regime. By breaking the mirror symmetry of the conventional M_2_XO_2_ architecture, the Janus configuration generates an intrinsic out-of-plane dipole and an internal electric field that redistribute charge unevenly between the two metal layers.^10,11^ The resulting electronic non-equivalence differentiates the two O-termination sites, creating a site-selective adsorption landscape that is inaccessible in symmetric MXenes.^10^ In this context, the site-resolved d-band centre (*ε*_d_)—computed separately for each metal sublattice—regains its utility as a mechanistic descriptor: it captures the very asymmetry that the Janus structure introduces and, as we show below, correlates strongly with the site-dependent Δ*G*_H*_ in non-magnetic systems.^6,12^ Out-of-plane ordered double-M MAX precursors with two early-d metals on the M-sublattice (*e.g.*, Mo_2_TiAlC_2_, Mo_2_NbAlC_2_) are well established experimentally,^8^ providing a chemically plausible route to ordered V/Nb Janus monolayers *via* selective etching.

Despite this promise, systematic studies of early d-metal Janus pairs remain limited. Most computational work on bimetallic MXenes has focused on late-transition-metal dopants or on structurally distinct ordered double-transition-metal carbides (*e.g.*, Mo_2_TiC_2_O_2_, Mo_2_NbC_2_O_2_).^7,12^ For the early d-metals of Groups 4, 5, and 6—which are earth-abundant and whose d-orbital chemistry is most amenable to systematic variation—a controlled comparison across metal pairs and, critically, across anion sublattices (carbide *versus* nitride) has not been reported. A complementary ML + DFT screen of ∼4500 M_1_M_2_XT_2_ compositions at the metal-top adsorption site identifies Nb/Mo/Cr-based O-terminated systems as promising,^13^ consistent with our identification of the VNb pair from a much narrower factorial. Our analysis is complementary in treating O-site adsorption and in resolving the spin state explicitly—necessary to capture the exchange-driven failure of the descriptor in CrMoNO_2_. Whether the anion identity plays a subordinate or decisive role in modulating HER activity in these systems is therefore an open question.

Here we address this gap through a spin-polarised GGA + *U* density functional theory study of six O-terminated bimetallic Janus MXenes, M_1_M_2_XO_2_ (M_1_/M_2_ = Ti/Zr, V/Nb, Cr/Mo; X = C, N), designed as a 3 × 2 factorial series. By pairing metals from the same periodic group, we hold the valence electron count fixed and isolate the influence of 3d/4d orbital spatial extent; varying the anion then provides an orthogonal compositional axis governed by electronegativity. We evaluate thermodynamic stability through formation energies, characterise the magnetic ground states, quantify Janus electronic asymmetry *via* site-resolved Bader charges and d-band centres, and map the hydrogen adsorption landscape at all inequivalent surface sites. Our results reveal that only the group-5 VNb pair achieves near-thermoneutral hydrogen binding: VNbCO_2_ (Δ*G*_H*_ = −0.03 eV) and VNbNO_2_ (−0.11 eV) both satisfy |Δ*G*_H*_| ≤ 0.3 eV, with the anion sublattice acting as a fine-tuning lever that adjusts binding strength within the thermoneutral window without destabilising the electronic structure. These findings establish computational compositional criteria—intermediate d-filling, quenched magnetism, and 3d/4d spatial asymmetry—for the rational design of Janus MXene HER electrocatalysts.

## 2 Computational methods

### 2.1 DFT setup and structural model

All calculations were performed using spin-polarised density functional theory (DFT)^14^ with the projector-augmented wave (PAW) method^15^ as implemented in the Vienna *Ab initio* Simulation Package (VASP).^16–19^ Exchange–correlation effects were described by the Perdew–Burke–Ernzerhof (PBE) functional within the generalised gradient approximation (GGA).^20^ To correct for the self-interaction error of localised d-electrons, the rotationally invariant GGA + *U* scheme of Dudarev *et al.*^21^ was applied to every transition-metal site. The effective Hubbard parameters (*U*_eff_, in eV) were: Ti 5.0,^22^ Zr 3.2,^23^ V 3.4,^24^ Nb 1.0,^25^ Cr 4.0,^26^ and Mo 2.0.^27^ A plane-wave kinetic-energy cutoff of 520 eV was employed; convergence tests confirmed that increasing the cutoff to 600 eV changed total energies by less than 1 meV per atom.

Each M_1_M_2_XO_2_ Janus MXene (M_1_/M_2_ = Ti/Zr, V/Nb, or Cr/Mo; X = C or N) was modelled as a periodic monolayer with a vacuum spacing of at least 11 Å along the *z*-axis to decouple periodic images; a dipole correction was applied along *z* to eliminate residual electrostatic interactions arising from the Janus out-of-plane asymmetry. Brillouin-zone integrations used a Γ-centred 8 × 8 × 1 *k*-point mesh with first-order Methfessel–Paxton smearing^28^ (*σ* = 0.05 eV), verified to converge total energies to within 1 meV per atom relative to a denser 12 × 12 × 1 mesh. All atomic positions were relaxed until the Hellmann–Feynman forces fell below 0.01 eV Å^−1^. Spin polarisation was enabled throughout; initial magnetic moments of 2*µ*_B_ per transition-metal atom were allowed to relax self-consistently.

### 2.2 Formation energy

Thermodynamic stability was assessed through the formation energy per atom,1
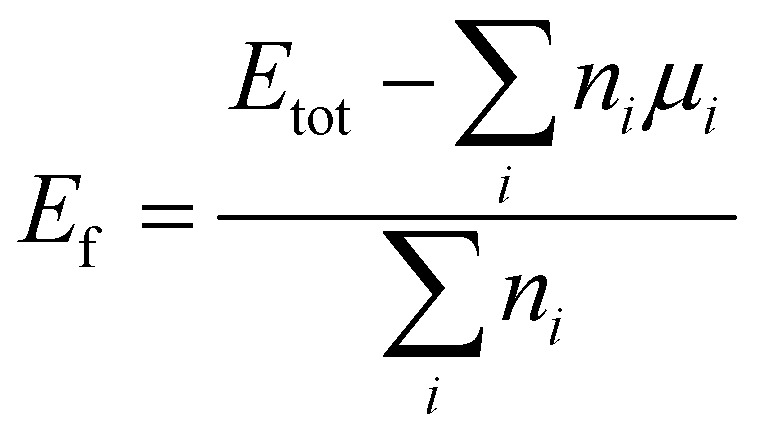
where *E*_tot_ is the DFT total energy of the relaxed Janus MXene unit cell, *n*_*i*_ is the number of atoms of species *i*, and *µ*_*i*_ is the chemical potential of species *i* taken from the DFT total energy per atom of the elemental standard state (bulk metal or the O_2_/N_2_/graphite reference, as appropriate). A negative *E*_f_ indicates stability against elemental decomposition.

### 2.3 Gibbs free energy of hydrogen adsorption

HER activity was evaluated within the computational hydrogen electrode (CHE) framework.^1^ Hydrogen adsorption was considered at four inequivalent surface sites: the O-termination above M_1_ (H@O(M_1_)), the O-termination above M_2_ (H@O(M_2_)), and the two metal top sites H@M_1_ and H@M_2_. The adsorption energy for each site is

2 Δ*E*_H*_ = *E*_slab+H_ − *E*_slab_ − ½*E*_H_2__,

where *E*_slab+H_ and *E*_slab_ are the total energies of the hydrogen-adsorbed and pristine slabs, and *E*_H_2__ is the total energy of a gas-phase H_2_ molecule computed in the same supercell (*E*_H_2__ = −6.72 eV). The Gibbs free energy of adsorption is

3 Δ*G*_H*_ = Δ*E*_H*_ + ΔZPE − *T*Δ*S*,

where ΔZPE is the zero-point energy correction and *T*Δ*S* the entropic contribution at *T* = 298 K. We adopt the standard empirical values ΔZPE = 0.04 eV and *T*Δ*S* = 0.40 eV,^1,29^ the latter reflecting the near-complete loss of translational and rotational entropy upon adsorption. This yields Δ*G*_H*_ = Δ*E*_H*_ − 0.36 eV. We note that explicit phonon calculations on the adsorbed state would refine these corrections, introducing an uncertainty of ∼0.1 eV;^1,30,31^ nonetheless, the empirical values are widely validated for comparative screening across structurally related systems.^2,29^ Site selection and HER candidacy are assessed independently. The preferred geometry is the configuration of minimum Δ*E*_H*_ (site stability, not catalytic optimality); HER candidacy then requires the corresponding Δ*G*_H*_ to satisfy |Δ*G*_H*_| ≤ 0.3 eV.^1,2,32,33^ TiZrCO_2_, TiZrNO_2_, and CrMoNO_2_ are stable but non-catalytic by this test. The full four-site tabulation is in Table S1.

### 2.4 Bader charge analysis

Site-resolved charge transfer was quantified by Bader charge analysis^34–36^ performed on the self-consistent charge densities of all six pristine Janus MXenes using the Henkelman group's grid-based decomposition algorithm.^34^ The VASP fine FFT grid (NGXF, NGYF, NGZF set to twice the default values) was used to ensure accurate integration of the Bader volumes. The net charge on each atom is defined as

4 Δ*q*_*i*_ = *q*^valence^_*i*_ − *q*^Bader^_*i*_

where *q*^valence^_*i*_ is the number of valence electrons in the PAW potential and *q*^Bader^_*i*_ is the integrated charge within the Bader volume. A positive Δ*q*_*i*_ indicates electron donation and a negative value indicates electron accumulation. These site-resolved charges provide a direct measure of the asymmetric charge redistribution between the M_1_ and M_2_ sublattices that defines the Janus electronic structure.

### 2.5 D-band centre

The d-band centre (*ε*_d_) was extracted separately for each metal sublattice from the site- and orbital-projected partial density of states (PDOS) obtained *via* the PAW projection scheme (LORBIT = 11). For each metal site, *ε*_d_ is the first moment of the spin-summed d-projected DOS relative to the Fermi level:5
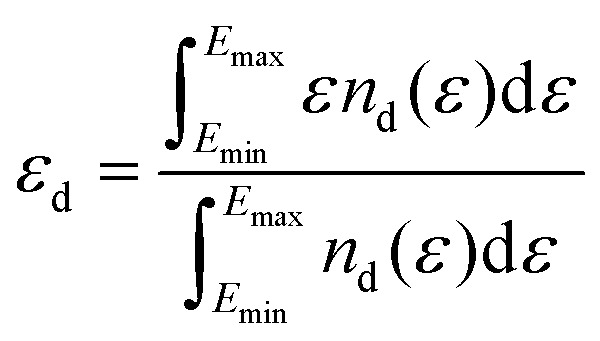
where *n*_d_(*ε*) = *n*^↑^_d_(*ε*) + *n*^↓^_d_(*ε*) is the spin-summed d-projected DOS at energy *ε* relative to *E*_F_.^37,38^ The integration window [*E*_min_, *E*_max_] = [−10, +6] eV relative to *E*_F_ was chosen to encompass the full d-band manifold; extending the window to [−15, +10] eV changed *ε*_d_ by less than 0.02 eV. The numerical integration was performed using a Python/NumPy script with a Fermi–Dirac broadening of 0.05 eV. This site-resolved approach is essential for Janus systems, where a single averaged *ε*_d_ would obscure the very electronic asymmetry that distinguishes the Janus structure from its symmetric counterpart.

## 3 Results and discussion

### 3.1 Crystal structure and thermodynamic stability

**Fig. 1:**
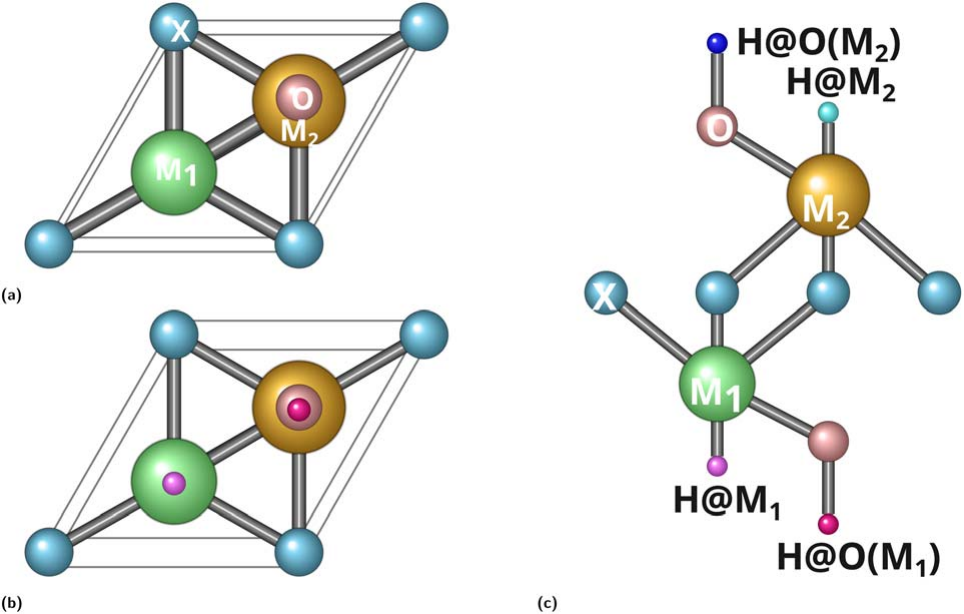
Crystal structures of the O-terminated bimetallic Janus MXenes (M_1_M_2_XO_2_): (a) pristine monolayer, (b) H-adsorbed (top view), and (c) H-adsorbed (side view). The five-layer stacking sequence O–M_1_–X–M_2_–O is common to all six systems; the broken mirror symmetry is evident from the inequivalent metal sublattices on opposite sides of the anion plane.

The optimised crystal structures of the O-terminated bimetallic Janus MXenes are illustrated in Fig. 1. Each monolayer adopts a hexagonal lattice with five atomic planes stacked along the *z*-direction in the sequence O–M_1_–X–M_2_–O, where M_1_ is the 3d metal (Ti, V, or Cr), M_2_ its 4d congener (Zr, Nb, or Mo), and X the anion (C or N). The two surface oxygen layers serve as the primary adsorption sites for hydrogen. Unlike conventional M_2_XO_2_ MXenes, the Janus configuration places chemically distinct metals on opposite sides of the anion plane, generating an intrinsic out-of-plane dipole that differentiates the two O-termination sites both structurally and electronically.

The optimised lattice parameters are summarised in Table 1. The in-plane lattice constant *a* contracts monotonically from the group-4 TiZr pair (*a* = 3.184 Å for TiZrCO_2_), through the group-5 VNb pair (3.040 Å), to the group-6 CrMo pair (2.974 Å), tracking the well-known contraction of atomic radii across the early transition-metal series. Replacing C with N generally contracts the lattice—*a* decreases from 3.184 to 3.112 Å in TiZr and from 2.974 to 2.935 Å in CrMo—owing to the shorter and stronger M–N bonds. The VNb system is a marginal exception (3.040 → 3.052 Å), where a subtle vertical registry rearrangement specific to the V/Nb pair slightly expands the basal plane in the nitride.

**Table 1:** Optimised in-plane lattice constant (*a*) and site-resolved net Bader charges (Δ*q*, in |*e*|) for the six Janus MXenes. Positive Δ*q* denotes electron donation; negative values denote electron accumulation. M_1_ and M_2_ are the 3d and 4d metal sites; X is the anion (C or N); O_M_1__ and O_M_2__ are the oxygen terminations adjacent to M_1_ and M_2_, respectively. The lattice contracts systematically from group 4 (TiZr) to group 6 (CrMo). In every system the 4d metal donates more charge than its 3d counterpart; this intra-Janus asymmetry increases upon C → N substitution in the VNb pair (from 0.14|*e*| to 0.19|*e*|)

System	*a* (Å)	Δ*q*(M_1_)	Δ*q*(M_2_)	Δ*q*(X)	Δ*q*(O_M_1__)	Δ*q*(O_M_2__)
TiZrCO_2_	3.184	+2.04	+2.22	−1.87	−1.17	−1.22
VNbCO_2_	3.040	+1.89	+2.03	−1.77	−1.03	−1.12
CrMoCO_2_	2.974	+1.68	+1.83	−1.52	−0.96	−1.03
TiZrNO_2_	3.112	+2.01	+2.22	−1.81	−1.18	−1.24
VNbNO_2_	3.052	+1.94	+2.13	−1.81	−1.10	−1.16
CrMoNO_2_	2.935	+1.76	+1.92	−1.63	−1.00	−1.05

The fractional *z*-coordinates confirm the strictly ordered five-layer vertical sequence in all systems, with unequal M_1_–X and X–M_2_ interlayer spacings. Taking CrMoCO_2_ as a structural prototype, the Cr–C spacing is noticeably shorter than the C–Mo spacing, consistent with the more compact 3d orbitals of Cr relative to the spatially extended 4d orbitals of Mo. This vertical asymmetry guarantees a built-in perpendicular dipole—the structural origin of the electronic non-equivalence between the two O-termination sites.

The thermodynamic stability of each Janus MXene, assessed through the formation energy per atom (*E*_f_, Table 2), spans a wide range. The group-4 systems are the most stable: TiZrCO_2_ (*E*_f_ = −2.76 eV per atom) and TiZrNO_2_ (−2.46 eV per atom) benefit from the highly electropositive character of Ti and Zr, which promotes strong ionic bonding. The group-5 systems occupy a moderate stability regime, with the carbide VNbCO_2_ (−0.85 eV per atom) more stable than the nitride VNbNO_2_ (−0.36 eV per atom)—consistent with the lower electronegativity of C (*χ*_C_ = 2.55) relative to N (*χ*_N_ = 3.04), which permits larger net charge transfer and stronger metal–anion bonding in the carbide. Among the group-6 systems, CrMoCO_2_ has a formation energy of −0.03 eV per atom, within DFT uncertainty and therefore not a reliable indicator of stability against elemental decomposition; we exclude it from the HER candidate set and retain it only as a non-magnetic data point for the Δ*G*_H*_ correlation (Section 3.6).

**Table 2:** Summary of computed properties for the most favoured Janus MXenes. *E*_f_: formation energy per atom (eV per atom); *µ*: magnetic moment (*µ*_B_ per cell); *E*_prist_: total energy of the pristine slab (eV); *E*_H@M_*i*__ and *E*_H@O(M_*i*_)_: total energies (eV) with H adsorbed at the M_*i*_ metal site or at the O-termination above M_*i*_; Δ*G*_H*_: Gibbs free energy of hydrogen adsorption (eV) at the most favourable site. Carbides are listed above the midline, nitrides below. VNbCO_2_ and VNbNO_2_ (bold) are the only systems satisfying |Δ*G*_H*_| ≤ 0.3 eV among those that are also thermodynamically stable (*E*_f_ < 0). The complete table for each configuration and corresponding Δ*G*_H*_ is given in SI

System	*E*_f_	*µ*	*E*_prist_ *E*_H@O(M_1_)_	*E*_H@O(M_1_)_	Δ*G*_H*_
TiZrCO_2_	−2.76	0.00	−44.80	−47.01	+0.79
VNbCO_2_	−0.85	0.01	−44.90	−47.93	−0.03
CrMoCO_2_	−0.03	−0.17	−43.65	−46.85	−0.20
TiZrNO_2_	−2.46	0.00	−46.49	−48.53	+0.96
VNbNO_2_	−0.36	0.08	−45.66	−48.77	−0.11
CrMoNO_2_	+0.34	2.67	−44.74	−47.27	+0.47

Crucially, the group-5 VNb systems—the principal pair of interest—are both thermodynamically stable (*E*_f_ < 0) and possess intermediate d-electron counts that, as shown below, place the O-termination electrophilicity in the optimal range for HER.

Formation energies against elemental references provide a necessary but not sufficient stability criterion. The standard next tier of validation—convex-hull analysis against competing phases plus phonon-dispersion or AIMD checks—is recommended in recent authoritative discussions.^39,40^ The present filter is intended to identify which members of the 3 × 2 factorial warrant that next-tier investment, and the VNb pair is the primary candidate so identified.

### 3.2 Magnetic properties

The calculated magnetic moments (Table 2) reveal a sharp group dependence. The group-4 systems (TiZrCO_2_, TiZrNO_2_) are entirely non-magnetic (0.00*µ*_B_), reflecting complete quenching of d-orbital moments by the strongly ionic Ti–O and Zr–O bonding. The group-5 systems are also effectively non-magnetic: VNbCO_2_ carries a negligible 0.01*µ*_B_ and VNbNO_2_ only 0.08*µ*_B_, both quenched by the crystal field and V/Nb d-state hybridisation with the X 2p and O 2p orbitals. A non-magnetic ground state is electronically favourable for HER because spin-degenerate channels at the Fermi level maximise the density of states available for charge transfer during the Volmer step.

The group-6 systems deviate sharply. CrMoNO_2_ carries a large moment of 2.67*µ*_B_, arising from the partially filled Cr d-shell that is insufficiently quenched by hybridisation with Mo. In contrast, CrMoCO_2_ shows only a weak antiparallel arrangement (−0.17*µ*_B_). This dichotomy reveals that the more electronegative N anion paradoxically preserves greater d-electron localisation on Cr, allowing exchange splitting to develop—a point confirmed by the spin-polarised density of states (Fig. 3), which shows that the moment is spatially localised on the Cr sublattice.

### 3.3 Charge transfer and Janus electronic asymmetry

The site-resolved Bader charges (Table 1) quantify the charge redistribution that defines the Janus electronic structure. Across all six systems both metal sites act as electron donors, transferring charge to the electronegative anion and surface oxygen layers. A universal pattern emerges: the 4d metal (M_2_) donates more charge than its 3d counterpart (M_1_) in every case. In VNbNO_2_, for instance, Nb donates +2.13|*e*| compared with +1.94|*e*| for V, yielding an intra-Janus asymmetry of Δ(Δ*q*) = 0.19|*e*|. This difference arises because the spatially extended 4d orbitals of Nb overlap more effectively with the anion and oxygen ligands than the compact 3d orbitals of V, facilitating greater charge transfer despite the near-identical Pauling electronegativities of V (*χ* = 1.63) and Nb (*χ* = 1.60). The same trend holds for TiZr (0.18|*e*| in the carbide, 0.21|*e*| in the nitride) and CrMo (0.15 and 0.16|*e*|).

Anion substitution (C → N) systematically increases the total charge accepted by the anion and oxygen sublattice, consistent with the higher electronegativity of N. In the VNb pair, Δ*q*(N) = −1.81|*e*| exceeds Δ*q*(C) = −1.77|*e*|, and the oxygen terminations also accumulate slightly more charge in the nitride. Importantly, the intra-Janus charge asymmetry itself increases upon C → N substitution in the VNb pair (from 0.14|*e*| to 0.19|*e*|), demonstrating that the more electronegative anion *amplifies* the Janus electronic non-equivalence. This amplification differentiates the two O-termination sites more strongly, creating a more favourable adsorption environment at the O(M_1_) site—a key factor in the superior HER performance of VNbNO_2_, as demonstrated below.

### 3.4 Site-dependent hydrogen adsorption

Hydrogen was placed at four inequivalent surface sites on each Janus MXene: H@O(M_1_), H@O(M_2_), H@M_1_, and H@M_2_. The total energies and corresponding Δ*G*_H*_ values are collected in Table 2. A volcano version showing both H@O(M_1_) and H@O(M_2_) configurations per system is provided in the supplementary information (Fig. S1).

A notable structural finding is that in VNbCO_2_ and VNbNO_2_, hydrogen initially placed on either metal site relaxes spontaneously onto the O-termination during geometry optimisation, without any imposed constraint; in CrMoCO_2_ the same occurs for H@M_2_. For these systems the O-site configuration is not merely the thermodynamic minimum but the *only* stable adsorption geometry accessible from the respective metal-site starting point. In the remaining systems (TiZrCO_2_, TiZrNO_2_, and CrMoNO_2_), H@M_1_ and H@M_2_ are locally stable but lie well above the H@O energy, confirming the general dominance of O-termination adsorption.^2,7^

For VNbNO_2_, the preferred H@O(M_1_) configuration yields Δ*G*_H*_ = −0.11 eV—well within the thermoneutral window. The H@O(M_2_) site lies 0.32 eV higher (Δ*G*_H*_ = +0.21 eV), a splitting that directly reflects the Janus electronic asymmetry: the V site (M_1_) donates less charge than Nb (M_2_), leaving the O-termination above V more electron-deficient and hence more electrophilic—the precise environment that strengthens the O–H bond toward thermoneutrality.

The contrast with VNbCO_2_ isolates the role of the anion sublattice. In the carbide, the lowest-energy O-site gives Δ*G*_H*_ = −0.03 eV, essentially thermoneutral within the uncertainty of the empirical thermal correction (∼0.1 eV). Replacing C with N shifts Δ*G*_H*_ by −0.08 eV (from −0.03 to −0.11 eV), moving the system further into the exothermic direction while keeping both members of the VNb pair within the low-overpotential regime. The more electronegative N draws additional electron density from the V/Nb bilayer, slightly enriching the O-termination and strengthening the O–H interaction.

The group-4 and group-6 systems bracket this optimum on either side. TiZrCO_2_ (+0.79 eV) and TiZrNO_2_ (+0.96 eV) under-bind hydrogen: the highly electropositive Ti/Zr metals donate so much charge to the anion sublattice that the O-termination becomes electron-saturated, leaving insufficient electrophilicity to hold H. Counterintuitively, the nitride is *more* under-binding (+0.17 eV shift), because the stronger charge withdrawal by N further depletes the residual metal-layer capacity to activate the surface O.

On the other flank, CrMoCO_2_ (−0.20 eV) also satisfies |Δ*G*_H*_| < 0.3 eV. However, its marginal thermodynamic stability (*E*_f_ = −0.03 eV/atom) and the presence of a competing H@O(M_2_) site at −0.13 eV—indicating multiple energetically similar configurations that may limit catalytic selectivity—make it a less robust candidate than the VNb pair. CrMoNO_2_, despite its large magnetic moment and the *a priori* expectation of stronger H binding, shows Δ*G*_H*_ = +0.47 eV: the exchange-split Cr d-states paradoxically over-donate charge to the anion sublattice, reducing surface electrophilicity and pushing the system into under-binding territory. The analysis uses the gas-phase isolated-H limit at the CHE level, the established screening protocol for O-terminated MXenes.^1,2,12,13^ Solvation and finite-coverage refinements^41–43^ are the appropriate next layer for the VNb pair specifically.

### 3.5 Volcano analysis and compositional trends

**Fig. 2:**
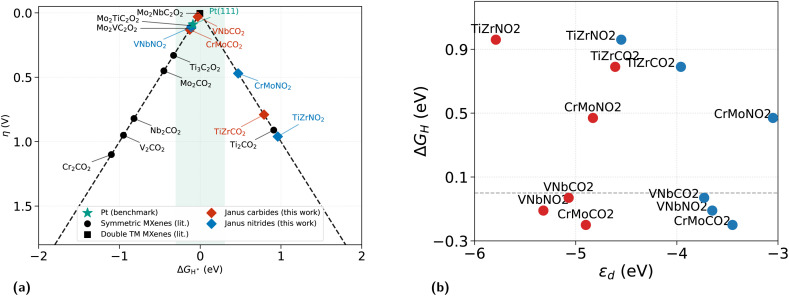
Variation of Δ*G*_H*_ with | Δ*G*_H*_| and *ε*_d_. (a) Theoretical HER volcano plot showing the overpotential *η* = |Δ*G*_H*_| as a function of Δ*G*_H*_ for the six Janus MXenes (filled symbols), alongside literature benchmarks for symmetric O-terminated MXenes (open circles) and ordered double-transition-metal MXenes (open squares). Pt(111) (red star) is shown for reference. The shaded band marks the thermoneutrality window |Δ*G*_H*_| ≤ 0.3 eV. Both VNbCO_2_ (−0.03 eV) and VNbNO_2_ (−0.11 eV) fall inside the window; CrMoCO_2_ (−0.20 eV) also satisfies the criterion but is marginally stable (see text). (b) Δ*G*_H*_versus pristine d-band centre *ε*_d_ for the 3d (M_1_, blue) and 4d (M_2_, red) metal sites. Each system appears as a horizontally separated pair at a common Δ*G*_H*_; the horizontal gap quantifies the intra-Janus *ε*_d_ asymmetry. The dashed line marks Δ*G*_H*_ = 0. Among the five non-magnetic or weakly magnetic systems, the 3d *ε*_d_ correlates strongly with Δ*G*_H*_ (*R* = −0.91); CrMoNO_2_ is a clear outlier due to exchange splitting (2.67*µ*_B_).

The computed Δ*G*_H*_ values are plotted on the volcano diagram in Fig. 2a. Three systems fall within the |Δ*G*_H*_| ≤ 0.3 eV window: VNbCO_2_ (−0.03 eV, *η* = 0.03 V), VNbNO_2_ (−0.11 eV, *η* = 0.11 V), and CrMoCO_2_ (−0.20 eV, *η* = 0.20 V). The two VNb systems occupy positions nearest the volcano apex, confirming the metal pairing as the decisive compositional variable. Although CrMoCO_2_ (−0.20 eV) lies nominally inside the window but is disqualified on stability grounds (Section 3.1) and retained only as a mechanistic data point.

Between VNbCO_2_ and VNbNO_2_, the carbide sits closer to the apex (|Δ*G*_H*_| = 0.03 *vs.* 0.11 eV), but the 0.08 eV difference is comparable to the inherent uncertainty in the empirical ΔZPE − *T*Δ*S* correction (−0.36 eV adopted here; literature values range from −0.24 to −0.40 eV depending on whether system-specific phonon frequencies are used).^1,29^ The salient point is therefore that *both* VNb systems lie within the low-overpotential window, whereas all other metal pairs—when combined with thermodynamic stability—do not. The two members represent complementary design targets: VNbCO_2_ for minimal overpotential, and VNbNO_2_ where a small exothermic driving force is desirable.

To understand why only the VNb pair achieves this, we trace the two compositional axes independently. Varying the metal pair at fixed N anion gives the progression Δ*G*_H*_ = +0.96 (TiZrNO_2_), −0.11 (VNbNO_2_), +0.47 eV (CrMoNO_2_)—a non-monotonic trend reflecting the competing effects of d-electron count and magnetism. The group-4 TiZr pair under-binds because excessive ionicity saturates the surface O; the group-6 CrMo pair in the nitride is unexpectedly *also* under-binding, because the 2.67*µ*_B_ exchange-split moment reduces the effective charge available to the O-termination. Only the group-5 VNb pair avoids both extremes.

Varying the anion at fixed metal pair quantifies the C/N tuning lever. For TiZr the shift is +0.17 eV (toward under-binding); for VNb it is −0.08 eV (deeper into the window); and for CrMo it is +0.67 eV—a large swing driven by the onset of exchange splitting in CrMoNO_2_. The anion substitution therefore does not act as a monotonic binding enhancer: its effect is mediated by the magnetic state of the metal pair. VNb is uniquely positioned as the combination where the anion produces only a modest, beneficial adjustment rather than a destabilising swing.

Compared with literature benchmarks (Fig. 2a), symmetric single-metal MXenes (Ti_2_CO_2_, V_2_CO_2_, Nb_2_CO_2_, Cr_2_CO_2_) all exhibit |Δ*G*_H*_| > 0.3 eV at their O-termination sites.^2,7^ The ordered double-transition-metal systems Mo_2_VC_2_O_2_ and Mo_2_NbC_2_O_2_ approach the apex through a structurally distinct three-layer carbide motif and achieve overpotentials comparable to VNbNO_2_.^12^ This equivalence is noteworthy: VNbNO_2_ matches the HER activity of these structurally complex systems using a simpler two-metal Janus nitride accessible *via* established synthetic routes for Janus monolayers.^10^

### 3.6 Electronic structure: d-band centre analysis and the origin of thermoneutrality

**Fig. 3:**
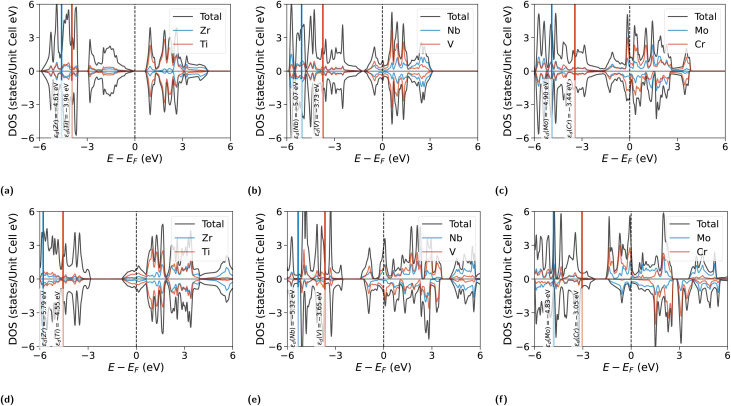
Spin-polarised total and site-projected partial density of states for the six Janus MXenes. Top row, carbides: (a) TiZrCO_2_, (b) VNbCO_2_, (c) CrMoCO_2_. Bottom row, nitrides: (d) TiZrNO_2_, (e) VNbNO_2_, (f) CrMoNO_2_. Black curves: total DOS; coloured curves: partial contributions from M_1_ (3d metal) and M_2_ (4d metal). The dashed vertical line marks *E*_F_ = 0; positive and negative DOS correspond to majority- and minority-spin channels. All six systems are metallic. In VNbNO_2_ (e) the V and Nb d-band contributions are clearly resolved near *E*_F_, with *ε*_d_(V) = −3.65 eV lying 1.67 eV above *ε*_d_(Nb) = −5.32 eV. CrMoNO_2_ (f) shows pronounced majority-spin spectral weight from exchange-split Cr d-states (2.67*µ*_B_). Vertical solid lines mark the site-resolved d-band centres *ε*_d_(M_1_) and *ε*_d_(M_2_), with values labelled adjacent.

The site-resolved d-band centres (Fig. 2b) and the spin-polarised DOS (Fig. 3) together provide the electronic foundation for the catalytic trends.

Within every system, the 4d metal (M_2_) exhibits a substantially deeper *ε*_d_ than the 3d metal (M_1_), with intra-Janus differences ranging from 0.65 eV (TiZrCO_2_) to 1.77 eV (CrMoNO_2_). This *ε*_d_ asymmetry is the electronic fingerprint of the Janus structure—absent by construction in symmetric MXenes—and provides the mechanism through which the two O-termination sites acquire distinct adsorption characteristics. For VNbNO_2_, the 1.67 eV separation between *ε*_d_(V) = −3.65 eV and *ε*_d_(Nb) = −5.32 eV generates the non-equivalent charge environments responsible for the 0.32 eV difference between H@O(M_1_) and H@O(M_2_) adsorption energies—a picture corroborated by the Bader charges (Table 1).

The effect of anion substitution on the d-band is most instructive within the VNb pair. Going from VNbCO_2_ to VNbNO_2_, *ε*_d_(Nb) shifts downward by 0.25 eV (from −5.07 to −5.32 eV), while *ε*_d_(V) shifts by only +0.08 eV (−3.73 to −3.65 eV, within the numerical integration uncertainty). The pronounced Nb downshift reflects the stronger charge withdrawal by N (*χ*_N_ = 3.04) from the spatially extended, more polarisable 4d orbitals; the compact 3d V orbitals are comparatively insensitive to the change in anion electronegativity. This selective depletion deepens *ε*_d_(Nb) and amplifies the charge asymmetry across the Janus bilayer, tipping the O(M_1_) termination—above the less-depleted V sublattice—into the electrophilic state that achieves Δ*G*_H*_ = −0.11 eV.

The CrMo pair shows an anomalous C → N trend. *ε*_d_(Cr) shifts *upward* by 0.40 eV (from −3.45 to −3.05 eV)—the opposite direction from the VNb behaviour. This inversion is a direct consequence of exchange splitting: the 2.67*µ*_B_ moment in CrMoNO_2_ separates the majority- and minority-spin Cr d-bands, pushing the spin-averaged *ε*_d_ upward. Far from indicating stronger H binding, this upshift is associated with *reduced* O-site electrophilicity and the positive Δ*G*_H*_ = +0.47 eV: the exchange-split d-band back-donates charge to the anion sublattice, saturating the surface O and weakening H capture. This constitutes a cautionary example: the d-band centre alone is not a sufficient descriptor in magnetically active Janus systems; the spin state must be treated as a co-descriptor alongside *ε*_d_.

Hydrogen adsorption uniformly upshifts *ε*_d_ by 0.3–0.8 eV at both metal sites across all systems, consistent with d-band narrowing as Fermi-level states are consumed by adsorbate bonding. The sole exception is Cr in CrMoNO_2_ (−0.07 eV downshift), attributable to exchange-splitting rearrangement. The consistency of this adsorption-induced upshift in all non-magnetic and weakly magnetic systems validates the pristine *ε*_d_ as the relevant descriptor for inter-system comparisons.

The correlation between pristine *ε*_d_ and Δ*G*_H*_ is visualised in Fig. 2b. For the 3d metal sites, a strong negative correlation (*R* = −0.91, excluding the magnetic CrMoNO_2_) connects *ε*_d_ to Δ*G*_H*_: deeper d-band centres correspond to weaker binding, reflecting reduced d-electron availability near *E*_F_ for activating the O-termination. The 4d metal sites show a weaker correlation (*R* = −0.18), consistent with the indirect influence of the subsurface M_2_ layer on the H-adsorbing O-site. CrMoNO_2_ deviates markedly on both panels—its Cr *ε*_d_ of −3.05 eV is the shallowest in the series, which by the non-magnetic trend should predict strong over-binding, yet its Δ*G*_H*_ is positive. This confirms that the spin-averaged *ε*_d_ loses its predictive power when significant spin polarisation is present. The detailed table is added in SI.

### 3.7 Mechanistic picture and design principles

The analyses above establish a coherent mechanistic picture. The VNb group-5 pair provides the appropriate d-electron count to place the O-termination near the optimal electrophilicity threshold. The anion sublattice acts as a fine-tuning lever within this pair: the more electronegative N selectively deepens *ε*_d_(Nb) and amplifies the Janus charge asymmetry (0.19|*e*| in the nitride *vs.* 0.14|*e*| in the carbide) without destabilising the electronic structure or triggering exchange splitting. Neither the TiZr pair (excessive ionicity) nor the CrMo pair (magnetic instability) can achieve this balance.

Three design criteria therefore emerge for Janus MXene HER catalysts: (i) an intermediate d-electron count (group 5) that avoids both excessive ionicity and exchange splitting; (ii) a quenched magnetic moment ensuring spin-degenerate Fermi-level channels for efficient charge transfer; and (iii) isoelectronegative yet spatially asymmetric 3d/4d metal sites that generate the charge non-equivalence necessary for site-selective O-termination adsorption.

The anion sublattice is a secondary handle in non-magnetic metal pairs (C → N shifts Δ*G*_H*_ by 0.08 eV in VNb), and a decisive one when it alters the surface magnetic state: in CrMo, C → N triggers a 2.67*µ*_B_ moment on Cr and a 0.67 eV swing that ejects the nitride from the thermoneutral window.

## 4 Conclusions

By screening six O-terminated bimetallic Janus MXenes across a controlled 3 × 2 factorial design, we have shown that a single compositional variable—the metal-pair identity—determines whether a Janus MXene can access the thermoneutral hydrogen-binding regime. Of the three same-group 3d/4d pairs examined, only the group-5 VNb combination satisfies both prerequisites for a viable HER electrocatalyst: thermodynamic stability (*E*_f_ < 0) and near-zero Δ*G*_H*_ (−0.03 and −0.11 eV for the carbide and nitride, respectively). The group-4 TiZr pair fails on the over-ionic side; the group-6 CrMo pair fails because exchange splitting in the nitride disrupts the d-band–Δ*G*_H*_ correlation that governs the non-magnetic systems (*R* = −0.91).

The origin of the VNb optimum is electronic rather than structural. Pairing metals from the same group holds the valence electron count fixed, so the Janus asymmetry arises entirely from the 3d/4d orbital-extent mismatch. This mismatch generates a site-resolved *ε*_d_ splitting of 1.67 eV, which differentiates the two O-termination sites and places one of them at the electrophilicity threshold required for thermoneutral H binding. The anion sublattice then provides an orthogonal tuning axis: C *versus* N shifts Δ*G*_H*_ by 0.08 eV within the VNb pair—a modest adjustment that keeps both systems inside the low-overpotential window—while the same substitution destabilises the CrMo pair by 0.67 eV through magnetic onset. The anion is therefore a secondary lever in non-magnetic pairs and a decisive variable when its substitution alters the surface magnetic state.

These findings distil into a concise design rule: optimal HER performance in Janus MXenes requires intermediate d-filling, quenched magnetism, and 3d/4d spatial asymmetry at matched electronegativity—conditions met uniquely by the VNb pair in the early d-metal series. We note that the empirical zero-point and entropy corrections used here carry an uncertainty of ∼0.1 eV; phonon calculations on the adsorbed state would sharpen the quantitative distinction between the carbide and nitride.

Experimental verification of V/Nb layering during selective etching and of V^4+^/Nb^4+^ preservation under HER conditions lies outside this computational screening and is required before the VNb pair can be advanced as a true synthetic target. Phonon-dispersion and constant-temperature AIMD validation of the VNb pair under HER conditions constitutes the principal computational next step. More broadly, the factorial screening strategy demonstrated here—orthogonal variation of metal pair and anion—offers a transferable template for navigating the high-dimensional compositional space of Janus MXenes toward targeted catalytic function.

## Author contributions

Shrestha Dutta: writing – original draft, visualization, validation, investigation, data curation. Rudra Banerjee: writing – review & editing, validation, supervision, methodology, formal analysis, conceptualization.

## Conflicts of interest

There are no conflicts to declare.

## Supplementary Material

RA-016-D6RA02989B-s001

## Data Availability

Data will be made available on request. Supplementary information (SI): Gibbs energy, Bader charge density and analysis of all 6 systems. See DOI: https://doi.org/10.1039/d6ra02989b.
