# Supplementary material for: Early-d-metal Janus MXenes as near-thermoneutral hydrogen evolution electrocatalysts: role of anion identity and d-band asymmetry
Source: RSC Adv. 2026 May 26;16(31):28507–15. doi: 10.1039/d6ra02989b (PMC13213557; doi:10.1039/d6ra02989b)
Supplement: RA-016-D6RA02989B-s001 [file RA-016-D6RA02989B-s001.pdf]

Supplementary Information (SI) for RSC Advances.
This journal is © The Royal Society of Chemistry 2026

Supplementary Information
Early-d-metal Janus MXenes as Near-thermoneutral Hydrogen Evolution Electrocatalysts: Role of Anion Identity and d-band Asymmetry

Shrestha Dutta, Rudra Banerjee*

Department of Physics and Nanotechnology, SRM Institute of Science and Technology, Kattankulathur, Tamil Nadu 603203, India

May 8, 2026

1 Site-resolved hydrogen adsorption energies

Table 1 reports the formation energy E_f, magnetic moment μ, pristine slab energy E_{prist}, and the H-adsorption energy E together with the corresponding ΔG_{H^*} at all four candidate adsorption sites (H@M_1, H@M_2, H@O(M_1), H@O(M_2)) for the six Janus MXenes. The data extend main-text Table 2 by exposing the energetic ordering of the four sites per system, making the two-step site-selection procedure described in main-text Section 2.3 (revised) auditable. The two HER candidates, $VNbCO_2$ and $VNbNO_2$, are highlighted in bold; for both, the configuration of minimum E corresponds to a hydrogen position at H@O(M_1). The superscript ^{a} marks initial metal-site placements that relaxed spontaneously to the nearest O-termination during structural optimisation, indicating that the metal-top sites are not locally stable on these systems and that O-site adsorption is the only physically realised configuration. The $CrMoNO_2$ entry is retained for completeness despite its disqualification as an HER candidate (positive E_f, magnetic ground state); it serves as the limiting case whose anomalous HER behaviour reveals the failure of the spin-averaged ε_d descriptor (main-text Section 3.5).

2 Charge density difference

Charge density differences $\Delta\rho = \rho(\text{slab} + \text{H}) - \rho(\text{slab}) - \rho(\text{H})$ at the preferred adsorption geometry H@O(M_1) for all six Janus MXenes are shown in Fig. 1. The two VNb systems (panels b, e) display the most pronounced spatial asymmetry of the family: cyan (depletion) lobes are sharply localised on V (M_1) with negligible features around Nb (M_2), and the yellow (accumulation) lobe is tightly concentrated on the O–H bond. This is the real-space signature of the Janus charge-redistribution asymmetry quantified integrally by the Bader charges in main-text Table 3—it is the V sublattice, not Nb, that supplies the charge consumed by the forming O–H bond, consistent with the placement of the most favourable adsorption site at O(M_1) and the asymmetric ε_d shifts on the two metal sublattices (main-text Section 3.5).

The TiZr systems (panels a, d) show diffuse, near-symmetric $\Delta\rho$ patterns, consistent with the over-ionic character that drives their under-binding behaviour: the system has already

*Email:rudrab@srmist.edu.in

System	E_f	μ	E_{prist}	H@M_1		H@M_2		H@O(M_1)		H@O(M_2)	
				E	ΔG_{H^*}	E	ΔG_{H^*}	E	ΔG_{H^*}	E	ΔG_{H^*}
$TiZrCO_2$	−2.76	0.00	−44.80	−45.84	1.96	−45.43	2.37	−47.01	0.79	−46.98	0.82
**$VNbCO_2$**	**−0.85**	**0.01**	**−44.90**	**−47.93**[a]	**−0.03**	**−46.28**[a]	**1.64**	**−47.93**	**−0.03**	**−47.84**	**0.06**
$CrMoCO_2$	−0.03	−0.17	−43.65	−45.11	1.54	−46.78[a]	−0.13	−46.85	−0.20	−46.78	−0.13
$TiZrNO_2$	−2.46	0.00	−46.49	−47.57	1.92	−47.35	2.14	−48.53	0.96	−48.26	1.23
**$VNbNO_2$**	**−0.36**	**0.08**	**−45.66**	**−48.77**[a]	**−0.11**	**−48.46**[a]	**0.19**	**−48.77**	**−0.11**	**−48.45**	**0.21**
$CrMoNO_2$	+0.34	2.67	−44.74	−46.22	1.52	−45.56	2.18	−47.27	0.47	−47.06	0.68

Table 1: Site-resolved hydrogen adsorption on the six Janus MXenes: formation energy E_f (eV/atom), magnetic moment μ (μ_B), pristine slab energy E_{prist} (eV), and adsorption energy E (eV) and ΔG_{H^*} (eV) at the four candidate sites: H@M_1, H@M_2, H@O(M_1), H@O(M_2). For each system the minimum-E configuration corresponds to the ΔG_{H^*} entry quoted in main-text Table 1. The superscript ^{a} marks initial metal-site placements that relaxed spontaneously to the nearest O-termination during structural optimisation; the listed energy is for the relaxed geometry, which coincides with H@O(M_i) (see main-text Section 3.4).

donated most of its available charge into the O termination in the pristine state, leaving little additional charge available for redistribution upon H adsorption. The CrMo systems (panels c, f) show comparatively weak isosurface activity at the chosen isovalue. In $CrMoNO_2$ (panel f) in particular, the small spatial $\Delta\rho$ is consistent with the 2.67 μ_B exchange-split state on Cr absorbing part of the H-adsorption response into spin-channel redistribution rather than purely spatial charge transfer—the same mechanism that breaks the spin-averaged ε_d descriptor in main-text Section 3.5.

3 *d*-band centre regression and statistical validation

The OLS regression of ΔG_{H^*} on the 3*d* (M_1) site-resolved *d*-band centre ε_d is computed across the five non-magnetic systems $TiZrCO_2$, $VNbCO_2$, $CrMoCO_2$, $TiZrNO_2$, and $VNbNO_2$; $CrMoNO_2$ is excluded on physical grounds—its 2.67 μ_B exchange-split *d*-band breaks the spin-averaged descriptor by construction, as discussed in main-text Section 3.5. The exclusion is the substantive scientific statement, not a data-cleaning operation: were $CrMoNO_2$ included, its residual against the non-magnetic fit would be +1.15 eV (Table 2), which is the quantitative signature of descriptor breakdown under exchange splitting rather than ordinary scatter. $CrMoCO_2$ is included in the regression as a non-magnetic, intermediate-*d*-electron-count data point independent of its disqualification as an HER candidate (main-text Section 3.1); the descriptor concerns electronic structure, not synthesizability, and the two assessments are independent.

Table 2 collects the full regression output: slope, standard error, intercept, residuals for each fitted point, and the corresponding 4*d* Pearson statistics for comparison. The 4*d* (M_2) coefficient ($R = -0.18$, $p = 0.73$) is statistically flat, consistent with the indirect coupling of the subsurface 4*d* layer to the H-adsorbing O-site. The wide 95% Fisher-*z* confidence interval $[-0.99, -0.12]$ on R reflects the unavoidable sampling uncertainty of a five-point fit and is reported explicitly rather than concealed; the load-bearing claim in the main text is the qualitative Hammer–Nørskov trend, not a tight quantitative fit.

4 Twelve-configuration volcano plot

The volcano plot in main-text Fig. 2(a) shows the minimum-E_{H^*} configuration per system (six markers). Fig. 2 presents the same data with separate markers for both H@O(M_1) and H@O(M_2) sites per system—twelve markers in total, paired by thin connecting lines to make

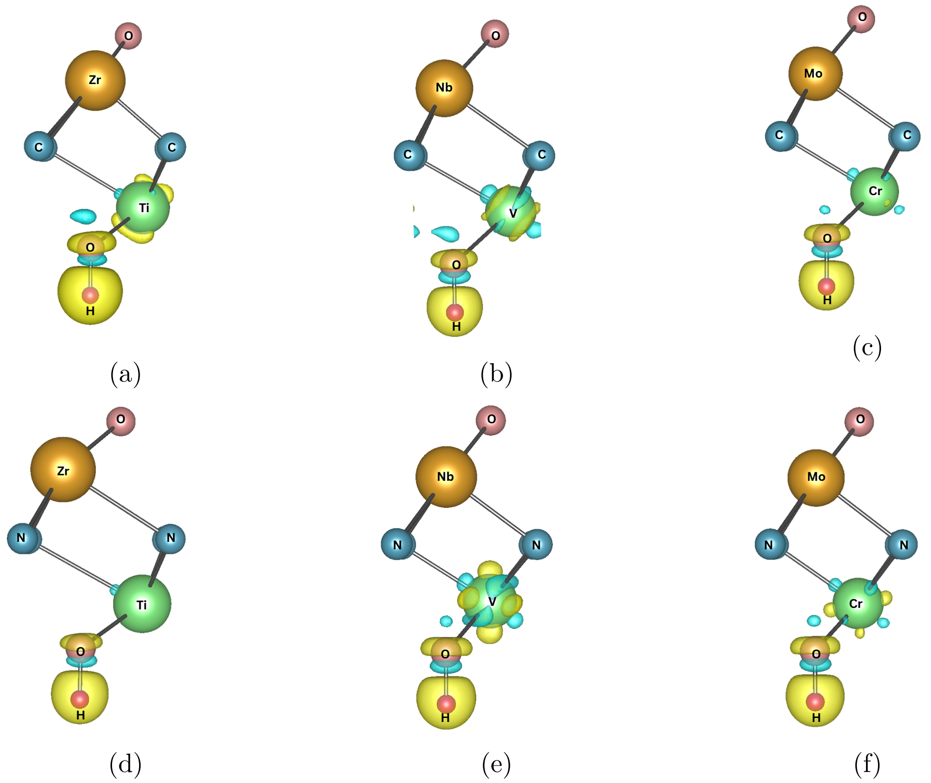

(a) (b) (c)

(d) (e) (f)

Figure 1: Charge density difference $\Delta\rho = \rho(\text{slab} + \text{H}) - \rho(\text{slab}) - \rho(\text{H})$ on H adsorption at H@O(M_1) for the six Janus MXenes. Top row, carbides: (a) $TiZrCO_2$, (b) $VNbCO_2$, (c) $CrMoCO_2$. Bottom row, nitrides: (d) $TiZrNO_2$, (e) $VNbNO_2$, (f) $CrMoNO_2$. Yellow isosurfaces denote charge accumulation, cyan isosurfaces charge depletion (isovalue $1 \times 10^{-3}\, e/\text{Å}^3$). The two VNb systems (b, e) show the most pronounced M_1-sided asymmetry; the magnetic $CrMoNO_2$ panel (f) shows weak spatial $\Delta\rho$ because part of the response is absorbed into spin-channel redistribution.

**Quantity**	**Value**
Pearson R	-0.91
R^2	0.82
p (two-tailed)	0.034
t-statistic (df = 3)	-3.70
95% Fisher-z CI on R	$[-0.99, -0.12]$
Slope	-1.17 eV/eV
SE (slope)	0.32 eV/eV
Intercept	-4.26 eV
Residuals (obs − pred)	
$TiZrCO_2$	+0.40 eV
$VNbCO_2$	−0.15 eV
$CrMoCO_2$	+0.01 eV
$TiZrNO_2$	−0.12 eV
$VNbNO_2$	−0.14 eV
$CrMoNO_2$ residual (excluded)	+1.15 eV
4d sites: R	-0.18
4d sites: p	0.73

Table 2: OLS regression statistics for ΔG_{H^*} vs. $\varepsilon_d(M_1)$ across the five non-magnetic 3d sites. The 4d Pearson R and p are provided for comparison. $CrMoNO_2$ is excluded on physical grounds (see main text Section 3.5); its residual against the non-magnetic fit (+1.15 eV) is the quantitative signature of descriptor breakdown under exchange splitting. On small-data correlation inference, see Refs. [1, 2, 3].

the intra-Janus splitting between the two O-termination configurations directly visible. The splitting is the catalytically relevant manifestation of the Janus electronic asymmetry discussed in main-text Sections 3.3 and 3.5: H@O(M_1) and H@O(M_2) sample two distinct local environments because the two metal sublattices ($M_1 = 3d$, $M_2 = 4d$) donate different amounts of charge to the surface oxygen layers, and it is this differential that produces the electrophilicity gradient required to bring the system toward thermoneutrality. The underlying numerical data are collected in Table 1 (columns H@O(M_1) and H@O(M_2)).

References

[1] Yuji Tomota, Kenji Nagasawa, and Takashi Kamachi. Automatic feature engineering for catalyst design using small data without prior knowledge of target catalysis. *Commun. Chem.*, 6:252, 2023.

[2] Bokinala Moses Abraham, Mullapudi V. Jyothirmai, Priyanka Sinha, Francesc Viñes, Jayant K. Singh, and Francesc Illas. Catalysis in the digital age: Unlocking the power of data with machine learning. *WIREs Computational Molecular Science*, 14(5):e1730, 2024.

[3] Jiaqi Chen, Junqing Li, Ziyi Liu, Shitao Sun, Shijia Zhou, and Dongqi Wang. Small-dataset-orientated data-driven screening for catalytic propane activation. *Artif. Intell. Chem.*, 3(1):100083, 2025.

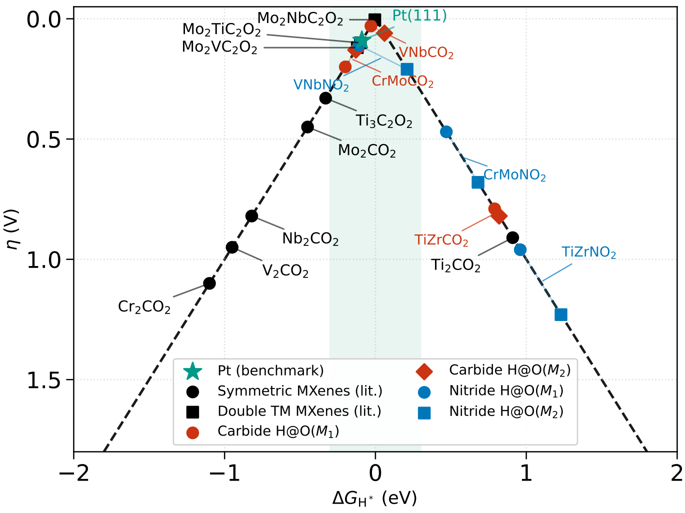

Figure 2: Volcano plot showing both H@O(M_1) and H@O(M_2) configurations for the six Janus MXenes (twelve markers, paired per system by thin connecting lines). The intra-Janus splitting between the two O-termination sites is the Janus electronic-asymmetry fingerprint discussed in main-text Sections 3.3 and 3.5. The shaded band marks the thermoneutrality window $|\Delta G_{\mathrm{H}^*}| \leq 0.3$ eV.
